# DPAGT1‐Mediated Protein *N*‐Glycosylation Is Indispensable for Oocyte and Follicle Development in Mice

**DOI:** 10.1002/advs.202000531

**Published:** 2020-06-03

**Authors:** Hui Li, Liji You, Yufeng Tian, Jing Guo, Xianbao Fang, Chenmin Zhou, Lanying Shi, You‐Qiang Su

**Affiliations:** ^1^ State Key Laboratory of Reproductive Medicine Nanjing Medical University Nanjing 211166 P. R. China; ^2^ Women's Hospital of Nanjing Medical University Nanjing Maternity and Child Health Hospital Nanjing Medical University Nanjing 211166 P. R. China; ^3^ Collaborative Innovation Center of Genetics and Development Fudan University Shanghai 200433 P. R. China; ^4^ Key Laboratory of Model Animal Research Nanjing Medical University Nanjing 211166 P. R. China

**Keywords:** DPAGT1, female infertility, meiosis, *N*‐glycosylation, oocyte quality

## Abstract

Post‐translational modification of proteins by *N*‐linked glycosylation is crucial for many life processes. However, the exact contribution of *N*‐glycosylation to mammalian female reproduction remains largely undefined. Here, DPAGT1, the enzyme that catalyzes the first step of protein *N*‐glycosylation, is identified to be indispensable for oocyte development in mice. *Dpagt1* missense mutation (c. 497A>G; p. Asp166Gly) causes female subfertility without grossly affecting other functions. Mutant females ovulate fewer eggs owing to defective development of growing follicles. Mutant oocytes have a thin and fragile zona pellucida (ZP) due to the reduction in glycosylation of ZP proteins, and display poor developmental competence after fertilization in vitro. Moreover, completion of the first meiosis is accelerated in mutant oocytes, which is coincident with the elevation of aneuploidy. Mechanistically, transcriptomic analysis reveals the downregulation of a number of transcripts essential for oocyte meiotic progression and preimplantation development (e.g., *Pttgt1*, *Esco2*, *Orc6*, and *Npm2*) in mutant oocytes, which could account for the defects observed. Furthermore, conditional knockout of *Dpagt1* in oocytes recapitulates the phenotypes observed in *Dpagt1* mutant females, and causes complete infertility. Taken together, these data indicate that protein *N*‐glycosylation in oocytes is essential for female fertility in mammals by specific control of oocyte development.

## Graphical abstract

Post‐translational modification is implicated in the control of gametogenesis. Through analysis of the infertile phenotype and its underlying mechanisms in female mice harboring either a missense point‐mutant allele or the oocyte‐specific knockout allele of *Dpagt1* gene, the indispensable role of protein *N*‐glycosylation in ensuring the development of high‐quality oocytes competent to complete meiotic division and support embryogesis is revealed.

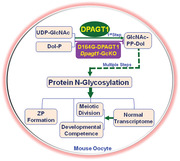

## 1 Introduction

The success of human reproduction relies on high quality sperm and oocytes.^[^ ^1^, ^2^ ^]^ Oocyte quality is manifested by the competence to complete meiosis, to be fertilized, and to support embryonic development.^[^ ^3^, ^4^ ^]^ This meiotic and developmental competence is gradually established during the course of oocyte and follicle development, and is determined in large part by the autonomous gene expression program intrinsic to the oocyte, albeit extrinsic factors present in follicular microenvironment, especially those committed in the oocyte–granulosa interaction is also crucial for oocyte development.^[^ ^3^, ^4^, ^5^, ^6^ ^]^ Despite that much has been learned about the control of oocyte development and gene expression, it still remains elusive as to how the oocyte quality is precisely determined.

Protein post‐translational modification (PTM), the final gatekeeper in the gene expression process, is crucial for the oocyte to build up the meiotic and developmental competence. For instance, oocyte meiotic arrest and progression, chromosome dynamics, and meiotic spindle assembly/disassembly are regulated by dynamic events of protein phosphorylation and dephosphorylation.^[^ ^7^, ^8^, ^9^ ^]^ Protein ubiquitination‐mediated degradation of CCNB1 and PTTG1 (also commonly known as Securin) is essential for metaphase‐to‐anaphase transition during oocyte meiotic maturation.^[^ ^10^, ^11^ ^]^ In addition, SUMOylation and de‐SUMOylation modifications orchestrated by members of the small ubiquitin‐related modifier (SUMO) protein family is required for oocyte meiotic resumption, proper kinetochore attachment to microtubules, and spindle formation during oocyte maturation.^[^ ^12^, ^13^, ^14^, ^15^, ^16^ ^]^ Moreover, other types of PTMs, such as protein methylation and acetylation, especially the histone family, and protein geranylgeranylation are also reported to be crucial for oocyte acquisition of meiotic and developmental competence, and the establishment of oocyte epigenome and oocyte–granulosa cell communication.^[^ ^17^, ^18^, ^19^, ^20^ ^]^ These protein PTMs add more layers of complexity to the control of oocyte development. Further deciphering the detailed downstream molecular effect of protein PTMs in oocytes will certainly provide valuable insights into the understanding of the sophisticated mechanism determining oocyte quality.

Protein glycosylation is one of the most frequent PTMs in eukaryotes, which play crucial roles in many life processes by impacting the structure and biological function of the proteins being modified.^[^ ^21^ ^]^ Two main types of glycosylation exist within the cells—*N*‐linked and *O*‐linked glycosylation, through which the specific glycan is covalently attached to the asparagine (Asn), and serine (Ser) or threonine (Thr) residues of the dedicated polypeptide backbone, respectively.^[^ ^21^ ^]^ Defects in the process of protein glycosylation can lead to many (>130) clinical diseases, collectively referred to as congenital disorders of glycosylation (CDG), which have multisystemic manifestations, e.g., developmental delay, failure to thrive, hypotonia, and neurologic abnormalities.^[^ ^22^ ^]^ Interestingly, in the female reproductive system, a large number of proteins are found to be glycosylated. These include several hormones and growth factors that are indispensable for oogenesis and folliculogenesis, e.g., the adenopituitary gland secreted gonadotropin FSH and LH, and their cognate receptors, oocyte‐derived paracrine factor GDF9 and BMP15, and granulosa cell secreted AMH.^[^ ^23^, ^24^, ^25^, ^26^ ^]^ This suggests that glycosylation process should be also critical for female reproduction, and fertility would be expected to be compromised in women with glycosylation disorders. However, few if any cases have been reported as to how glycosylation disorder may affect female reproduction in human. The exact role of protein glycosylation in the control of mammalian oogenesis and folliculogenesis, as well as the overall processes of female reproduction, still remains largely elusive. This could be due to the severity and infantile onset of the neuromuscular and other multisystem disorders that prevent the patients from surviving to adulthood or mask the fertility problem. Indeed, deletion of most if not all of the genes encoding the critical enzymes catalyzing glycosylation causes embryonic lethality in mice, which hinders further exploration of the role of glycosylation in female reproduction.^[^ ^27^ ^]^ Therefore, it is highly desirable to establish alternative animal models, such as point mutations that inactivate critical enzymes for glycosylation processes but leave gene products otherwise intact, and the *Cre‐LoxP*‐mediated conditional knockout (cKO) of these genes, to circumvent this barrier and to better decipher the role of glycosylation in the control of female reproduction.

Herein, we established two mutant alleles of mouse *Dpagt1* gene that encodes the critical enzyme committed in the first step of protein *N*‐glycosylation: an ethylnitrosourea (ENU) ENU‐induced whole‐body recessive missense point mutation, and the *Gdf9*‐Cre mediated oocyte‐specific knockout. By exploring the effect and underlying mechanism of these two types of *Dpagt1* mutation on female fertility, we uncovered an oocyte‐specific role of protein *N*‐glycosylation in ensuring the development of an oocyte with both meiotic and developmental competence.

## 2 Results

### 2.1 Oocyte Quality and Female Fertility Were Compromised in Mice Carrying a Missense Point‐Mutation of *Dpagt1*

**Figure 1:**
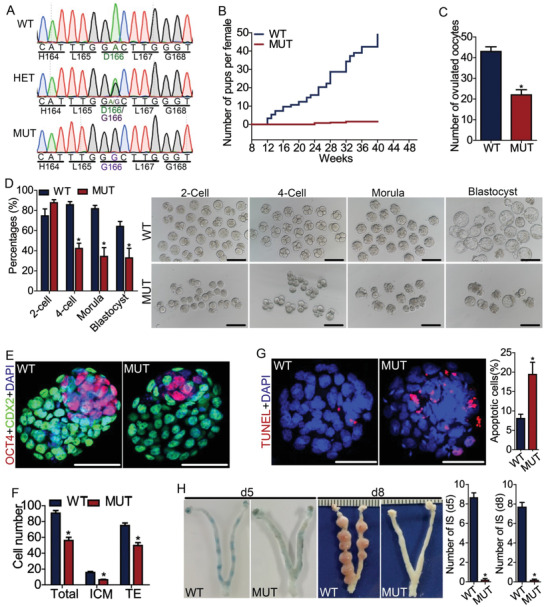
D166G‐*Dpagt1* missense point‐mutation compromises oocyte quality and female fertility in mice. A) Chromatogram of Sanger sequencing illustrating the ENU‐induced D166G‐missense point‐mutation of *Dpagt1*. WT, wild type; HET, heterozygous; MUT, mutant. B) Cumulating number of pups born per female by the WT (*n* = 4) and MUT (*n* = 4) females during the 8 month period of fertility test. C) Number of oocytes ovulated by WT and MUT females after superovulation treatment. D) The rate (left panel) and representative micrograph (right panel) of 2‐cell, 4‐cell, morula, and blastocyst formed by ovulated WT and MUT oocytes after IVF. Scale bars indicate 100 µm. E) IF staining of blastocysts derived from WT and MUT oocytes for OCT4 (red) and CDX2 (green), markers of inner cell mass (ICM) and trophectoderm (TE), respectively. DNA was counterstained with DAPI (blue). Scale bars: 50 µm. F) Quantification of the number of cells for ICM, TE, and all cell types following IF staining in (E). G) Terminal deoxynucleotidyl transferase‐mediated dUTP nick end labeling (TUNEL) staining of the blastocysts derived from WT and MUT oocytes. Left panel indicates the representative micrograph (scale bars = 50 µm); right graph showing the quantification of the positive cells. H) Assessment of the implantation potential of blastocysts derived from WT and MUT oocytes. Left panels are micrographs showing the implantation sites (IS) in the uteri at day 5 and day 8 postcoitus. Right graphs are the quantification of the IS. *p < 0.05, compared with WT by Student's *t*‐test.

ENU‐induced mutagenesis created an autosomal recessive point mutation of *Dpagt1* in mice. This A to G transition at nucleotide 497 of the coding region caused missense mutation of the 166th aspartic acid (D) into glycine (G), hereafter referred to as D166G mutation (**Figure** **1**A). This D166G mutation did not affect the expression of *Dpagt1* mRNA (Figure S1A, Supporting Information), nor did it affect the expression and localization of DPAGT1 protein in oocytes (Figure S1B, Supporting Information). However, it severely compromised the fertility of the females. D166G‐*Dpagt1* homozygous mutant (MUT) females were nearly infertile, with only an average of 1.5 pups per female produced during the entire period of 7 month fertility test (Figure 1B). The fecundity of mutant females differs dramatically from the wildtype (WT) littermate that yielded about 49 pups per female at the same testing period (Figure 1B).

*Dpagt1* was expressed at a medium level in the ovary and oocyte compared with all the other 8 different tissues examined (Figure S1C, Supporting Information), and within the follicles, it was expressed at a relatively lower level in the oocyte than in the follicular somatic cells, i.e., cumulus cells (CCs) and mural granulosa cells (MGCs) (Figure S1D, Supporting Information). Despite the higher expression of *Dpagt1* in some other key tissues (e.g., heart, lung, and liver) (Figure S1C, Supporting Information), D166G‐*Dpagt1* mutant mice survive well, and look grossly normal.

Oocyte quality is a key limiting factor for female fertility. ^[^ ^28^, ^29^ ^]^ The impairment of female fertility in D166G‐*Dpagt1* mutants could be attributed to changes of oocyte quality. This possibility was therefore tested by superovulation of the mutant females and in vitro fertilization of the eggs ovulated. As shown in Figure 1C, D166G‐*Dpagt1* mutant females could ovulate, but the yield was much lower than that of the WT littermates (22 oocytes per mouse in Mutants vs 43 oocytes per mouse in WTs) (Figure 1C). Eggs ovulated by D166G‐*Dpagt1* mutants could be fertilized and undergo the first round of cleavage normally (Figure 1D). However, further development of the 2‐cell stage embryos into blastocysts was severely impaired in D166G‐*Dpagt1* mutants, with the rate of blastocyst formation was only 33%, which is significantly lower than the 64% in WTs (Figure 1D).

Furthermore, the fewer blastocysts formed by D166G‐*Dpagt1* mutant oocytes through in vitro fertilization and embryo culture also appeared abnormal. Staining of these blastocysts with antibodies against OCT4 and CDX2, two cell lineage specific markers for inner cell mass (ICM) and trophectoderm (TE), respectively, demonstrated that the number of ICM and TE cells, as well as the total cell number, was significantly lower than that of WTs (Figure 1E,F). TUNEL staining demonstrated an evident increase of apoptosis in D166G‐*Dpagt1* mutant blastocysts, with the TUNEL‐positive cells in the mutant doubled those of the WT (Figure 1G). In accordance with the reduced cell content and increased apoptosis in the mutant blastocyst, implantation sites (ISs) were barely detected in the uteri of D166G‐*Dpagt1* mutants subjected to a timed mating scheme on either day 5 or day 8 postcoitus (Figure 1H).

### 2.2 Follicle Development Was Impaired in D166G‐*Dpagt1* Mutant Females

**Figure 2:**
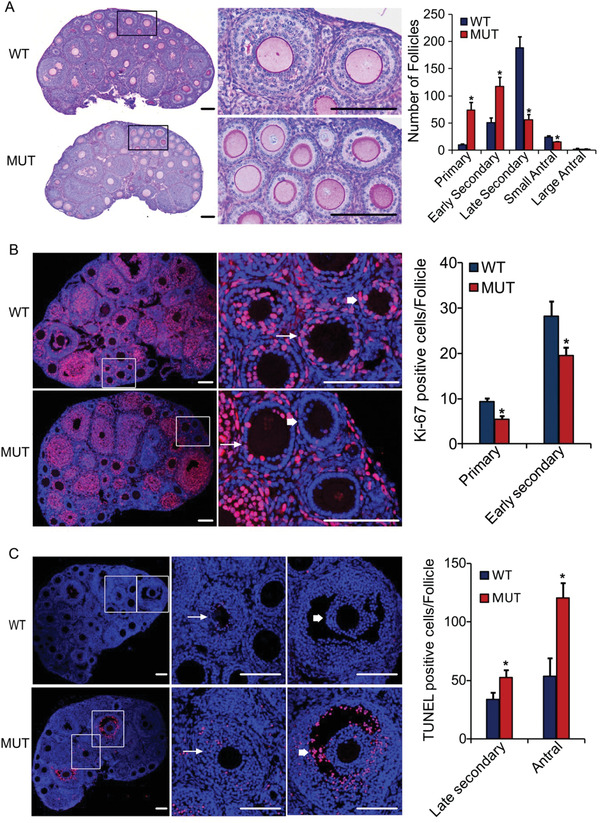
Defective follicular development in D166G‐*Dpagt1* MUT ovaries. A) The left panels are the representative microphotographs of 21 day old WT and MUT mouse ovarian sections stained with PAS and hematoxylin. The amplified views of the boxed area are shown on the right side. The right bar graph is the quantification of the number of follicles at different stages of development. B) IF staining of cell proliferation marker Ki‐67 (red) in the ovaries. The representative micrographs are shown in the left panels, with the amplified views of the boxed area shown on the right side. Thick arrows point to primary follicles, while thin arrows indicate early secondary follicles. The right graph is the quantification of the Ki‐67 staining. C) TUNEL staining (red) of granulosa cell apoptosis in the ovarian follicles. The representative micrographs are shown in the left panels, with the amplified views of the boxed area shown on the right side. Thick arrows point to antral follicles, while thin arrows indicate late secondary follicles. The right graph is the quantification of the TUNEL staining. DNA is counterstained with DAPI. Scale bars indicate 100 µm. *p < 0.05, compared to WTs by Student's *t*‐test.

The reduction in the number of ovulated oocytes in D166G‐*Dpagt1* mutant females suggests that follicular development might be impaired in these mice. In order to test this possibility, the first wave of follicular development was analyzed by counting the number of follicles at various stages of development on the 21 day old mouse ovarian serial sections that were stained with Periodic Acid‐Schiff (PAS) Stain. Dramatic differences were observed between the D166G‐*Dpagt1* mutant and WT mice in the number of preantral stage growing follicles. There were more primary and early secondary follicles present in the mutant ovaries, but the number of late secondary and small antral follicles was markedly reduced in the mutant (**Figure** **2**A). Nevertheless, no significant difference was observed between the WT and D166G‐*Dpagt1* mutant in the number of total and primordial follicles (Figure S2A, Supporting Information). The similar changes of preantral follicular development were also observed in the 21 day old D166G‐*Dpagt1* mutant females after priming with PMSG, with the exception of no significant changes in the early secondary follicles in the mutant (Figure S2B, Supporting Information). The number of large antral follicles was also found to be reduced in these PMSG‐primed mutant mice (Figure S2B, Supporting Information).

To decipher what causes the defects of follicular development in D166G‐*Dpagt1* mutant females, cell proliferation and apoptosis were analyzed by Ki‐67 and TUNEL staining, respectively, on 21 day ovarian sections. The most prominent differences in cell proliferation were found within the primary and early secondary follicles, where fewer Ki‐67 positive granulosa cells were present in the mutant than the WT (Figure 2B). As to TUNEL staining, changes were only detected in late secondary and antral follicles, with more TUNEL positive granulosa cells found in the mutant mouse ovaries (Figure 2C).

### 2.3 Formation of Zona Pellucida (ZP) and *N*‐Glycosylation of ZP Proteins Were Disrupted in Oocytes of D166G‐*Dpagt1* Mutant Females

**Figure 3:**
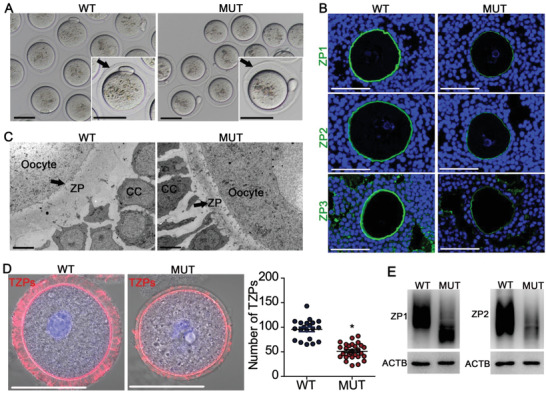
Defective formation of ZP coat and TZPs in oocytes of D166G‐*Dpagt1* mutant females. A) Light micrographs showing the thin and fragile ZP coat in ovulated MUT oocytes. Arrows point to ZP. Scale bars: 50 µm. B) IF staining of ZP1‐3 proteins (green) in GV‐stage immature oocytes in small antral follicles. DNA was counterstained by DAPI (blue). Scale bars indicate 50 µm. C) Transmission electron microscopic graphs of COCs. CC, cumulus cells. Scale bars indicate 5 µm. D) Phalloidin staining of TZPs (red) in immature GV‐oocytes. The left panel is the representative micrographs, and the right graph is the quantification of the number of TZPs per oocyte. Scale bars indicate 50 µm. *p < 0.05, compared to WTs by Student's *t*‐test. E) Western blot analysis of ZP1, ZP2, and ACTB expression in GV‐stage immature oocytes.

All vertebrate oocytes are coated with ZP, an extracellular matrix (ECM) composed of ZP‐glycoproteins. In mice, ZP starts to form in growing oocytes of primary follicles, and a complete ZP coat is essential for oocyte and follicle development. Given the glycosylation nature of ZP‐proteins, the potential influence of *Dpagt1* mutation on ZP formation was investigated. Indeed, ZP coat of the mature oocytes ovulated by D166G‐*Dpagt1* mutants was thinner and more fragile, and the perivitelline space in these oocytes appeared wider than that of the WT oocytes (**Figure** **3**A). Thinner ZP was also observed in the immature growing (Figure 3B) and fully grown oocytes (Figure 3C) by immunofluorescece (IF) staining of the three components of ZP (i.e., ZP1, ZP2, and ZP3) on ovarian sections and transmission electron microscopy analysis of the oocyte‐cumulus complexes, respectively.

In the ovarian follicles starting from the growing stage, the connection between the oocyte and granulosa cells is established by a delicate structure termed transzonal projection (TZP), which is a specialized cytoplasmic extension of granulosa cells that traverses the ZP and reach the oocyte plasma membrane. The flawed ZP structure in D166G‐*Dpagt1* mutant oocytes might impair TZPs and the connection between the oocyte and granulosa cells, hence interfering oocyte–granulosa cell communication. To test this possibility, the distribution of TZPs was compared between D166G‐*Dpagt1* mutant and WT oocytes. Since most TZPs contain a central core of actin, TZPs were monitored by staining the oocyte with the F‐actin‐binding dye, phalloidin, followed by counting the number of actin bundles within the ZP. As shown in Figure 3D, sparsed distribution of F‐actin filaments was detected in the ZP‐zone of D166G‐*Dpagt1* mutant oocytes, which differs significantly from the dense pattern of distribution in the WT oocytes.

Consistent with the aforementioned morphological defect of the ZP coat, western blot analysis revealed apparent downshift in the molecular mass of ZP1 and ZP2 proteins, as well as the reduction of the total amount of ZP2 protein in D166G‐*Dpagt1* mutant oocytes (Figure 3E). This observation is indicative to the reduction of protein glycosylation of the ZP proteins. In order to ascertain whether these changes in ZP proteins are indeed attributed by alterations in their status of *N*‐glycosylation, and to unravel the more broad effect of D166G‐*Dpagt1* mutation on protein *N*‐glycosylation in the ovary, high‐accuracy mass spectrometry‐based *N*‐glycoproteomic analysis was carried out on the D166G‐*Dpagt1* mutant ovaries. This resulted in the detection of a total of 2169 and 2213 glycosites in the WT and the mutant ovaries, respectively, which mapped to 2703 and 2743 glycopeptides, and 1051 and 1071 glycoproteins in the corresponding WT and mutant ovaries. By applying the criteria of *p* < 0.01 and fold change >2, *N*‐glycosylation in 45 proteins was identified to be downregulated (Figure S3A and Table S2, Supporting Information), and that in 13 proteins was upregulated in the D166G‐*Dpagt1* mutant ovaries (Figure S3A and Table S3, Supporting Information). Enrichment analysis revealed that the up‐ and downregulated glycoproteins were involved in distinct biological processes and pathways (Figure S3B, Supporting Information). Notably, glycosylation of all three ZP proteins was found to be significantly downregulated in D166G‐*Dpagt1* mutant ovaries, with the fold change of −17 355.8/−4.9, −16 829.5, and −4.6 for ZP2, ZP3, and ZP1, respectively (Figure S3A and Table S2, Supporting Information).

### 2.4 Oocyte Transcriptome Was Distorted in D166G‐*Dpagt1* Mutant Females

**Figure 4:**
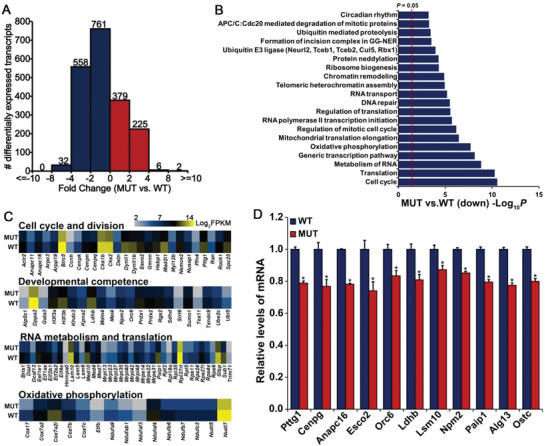
Distortion of the oocyte transcriptome in D166G‐*Dpagt1* mutant oocytes. A) Distribution of the significantly changed transcripts at various magnitudes of difference in expression levels between MUT‐ and WT‐GV‐stage immature oocytes detected by RNA‐Seq. The number of changed transcripts in each category of fold change is indicated above each bar. B) Bar graph illustrating the enriched GO/KEGG terms or canonical pathways associated with the significantly downregulated transcripts in MUT GV‐stage oocytes identified by RNA‐Seq. C) Heatmaps illustrating differences between WT and MUT GV‐stage immature oocytes in the expression of a cohort of transcripts involved in various processes. D) Quantitative real‐time RT‐PCR validating changes in representative transcripts selected from RNA‐Seq data. *p < 0.05, compared to WTs by Student's *t*‐test.

To unravel the potential molecular mechanisms attributing to the defective changes in the oocyte and follicle of D166G‐*Dpagt1* mutant‐females, transcriptomic analysis was carried out on the GV‐stage immature FGOs of D166G‐*Dpagt1* mutant and WT mice. A total of 1963 transcripts were identified to be significantly changed in the D166G‐*Dpagt1* mutant oocytes as compared with WTs, of which 1351 transcripts were downregulated and 612 were upregulated, respectively, in the mutant oocytes (**Figure** **4**A; Tables S4 and S5, Supporting Information). Gene enrichment analysis revealed that the downregulated transcripts were mainly associated with processes related to “cell cycle and cell division,” “mRNA metabolism and translation,” and “oxidative phosphorylation” (Figure 4B), which differs from those enriched by the upregulated transcripts in D166G‐*Dpagt1* mutant oocytes (Figure S4, Supporting Information). Changes in the expression of the transcripts in these key processes, as well as those reported to be crucial for oocyte meiotic and developmental competence are shown in Figure 4C. Downregulation of the representative transcripts in these key processes and the pathway of protein *N*‐glycosylation were validated by real‐time quantitative RT‐PCR (qRT‐PCR) (Figure 4D).

### 2.5 Fidelity of Oocyte First Meiotic Dividion Was Skewed in D166G‐*Dpagt1* Mutant Females

**Figure 5:**
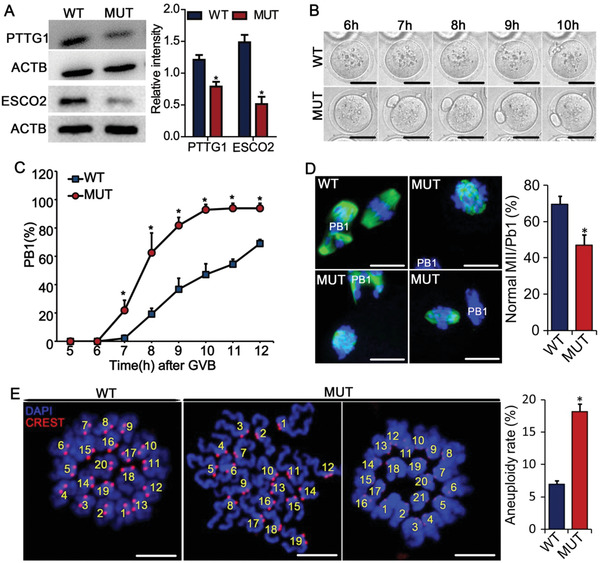
Defective progression and completion of the first meiosis in D166G‐*Dpagt1* mutant oocytes. A) WB analysis of PTTG1, ESCO2, and ACTB expression in GV‐stage immature oocytes. B) Representative still images from the spinning disk confocal live imaging of PB1 extrusion by oocytes in culture. Time indicates hours after oocytes being released from meiotic arrest. Scale bars dictate 50 µm. C) Kinetics of PB1 extrusion in oocytes after being released from meiotic arrest and matured in culture. *p < 0.05, compared to WTs at the same timepoint by Student's *t*‐test. D) Confocal microscopic analysis of spindle morphology and chromosome alignment in mature oocytes. Representative micrographs of IF staining of tubulin (green) and chromosome (blue) are shown on the left. Scale bars indicate 10 µm. Quantification of the rate of oocytes reaching normal MII is shown on the right. E) Aneuploidy assessment in mature oocytes. Left panels are the representative micrographs of the spread chromosome. Centromeres are stained in red with CREST antibody, and chromosomes are stained in blue by DAPI. The right graph is the quantification of the incidence of aneuploidy detected in (A). Scale bare indicate 10 µm. *p < 0.05, compared to WTs by Student's *t*‐test.

Transcriptomic analysis in the preceding experiment revealed that the expression of *Pttg1* and *Esco2* was downregulated in D166G‐*Dpagt1* mutant oocytes (Figure 4C,D). The downregulation of *Pttg1* and *Esco2* expression was confirmed at both the mRNA (Figure 4D) and protein (**Figure** **5**A) levels by qRT‐PCR and western blot analyses, respectively. Given that PTTG1 and ESCO2 are essential for ensuring the precision timing of meiotic progression and accuracy of chromosome segregation,^[^ ^10^, ^30^ ^]^ the reduction of PTTG1 and ESCO2 in D166G‐*Dpagt1* mutant oocytes might interfere with the normal kinetics of meiotic progression and the faithful separation of the homologous chromosomes in oocytes. To test this possibility, time‐lapse live imaging of the oocytes undergoing in vitro maturation (IVM) was conducted by spinning disc confocal microscopy. Indeed, completion of the first meiosis was significantly accelerated in D166G‐*Dpagt1* mutant oocytes as judged by the precocious extrusion of the first polar body (PB1) (Figure 5B,C). In WT oocytes, PB1 extrusion took place at 8–10 h after germinal vesicle breakdown (GVB, the indication of oocyte resumption of the first meiosis), whereas in the mutant oocytes, it occurred at 7 h post‐GVB, which was 1–2 h earlier than in the WT oocytes (Figure 5B,C). IF staining of microtubules and chromosomes in the oocytes with extruded PB1 revealed that the percentage of oocytes at normal metaphase II (MII) stage was significantly lower in D166G‐*Dpagt1* mutants (69.7% in WTs vs 47.0% in mutants), and the mutant oocytes that were not at normal MII stage displayed aberrant spindles and misaligned chromosomes (Figure 5D). Chromosome spread analysis of the mature oocytes demonstrated that the rate of aneuploidy was dramatically elevated in D166G‐*Dpagt1* mutants (Figure 5E).

**Figure 6:**
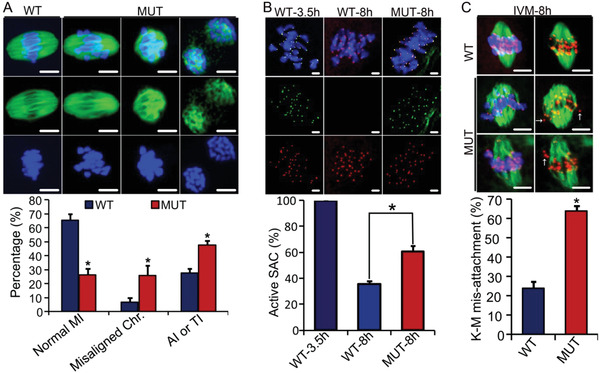
Defective progression of meiosis to metaphase I (MI) by D166G‐*Dpagt1* mutant oocytes. A) IF staining of spindles (green) and chromosomes (blue) in oocytes matured in vitro for 8 h (top panel). Quantification of the rate of oocytes with various meiotic behaviors is shown in the bottom panel. B) Assessment of the active status of SAC by IF staining of BUBR1 (green) and kinetochore (red) in oocytes (top panel). Normal WT oocytes matured in vitro for 3 h serve as positive control for active SAC. Quantification of the percentage of oocytes with active SAC is shown in the bottom bar graph. C) Analysis of kinetochore–microtubule attachment in oocytes matured in vitro for 8 h. Microtubules are stained in green, kinetochores are stained in red, and chromosomes are stained blue. Quantification of the percentage of oocytes with misattached kinetochore–microtubules are shown in the bottom panel. Arrows indicate chromosomes with no microtubule attached. Scale bars indicate 5 µm. *p < 0.05, compared to WTs by Student's *t*‐test.

Precocious completion of the first meiosis observed in D166G‐*Dpagt1* mutant oocytes suggests that the control of the progression of the first meiosis might go awry. This possibility was therefore tested by assessing oocyte competence of progression of meiosis to metaphase I (MI). IF staining of the spindles and chromosomes in the oocytes that have been cultured in maturation medium for 8 h revealed significant differences between the WT and the mutant (**Figure** **6**). Unlike in WTs where 65.6% of the oocytes were at normal MI stage with normal spindles and well‐aligned chromosomes, only 26.4% of oocytes in D166G‐*Dpagt1* mutants were at normal MI stage (Figure 6A). There was a significantly larger proportion of D166G‐*Dpagt1* mutant oocytes either having abnormal MI spindles with misaligned chromosomes associated, or already reaching the anaphase I (A I) or telophase I (T I) (Figure 6A). Consistent with the presence of misaligned chromosomes in D166G‐*Dpagt1* mutant oocytes, IF staining of the spindle assembly checkpoint (SAC) protein BUBR1 revealed that there were more mutant oocytes stained positively for BUBR1 at the centromeric region (Figure 6B), indicative of the defect in timely inactivation of SAC. In accordance with the presence of SAC defects, there was a dramatic increase in the errors of kinetochore–microtubule (K–M) attachment in D166G‐*Dpagt1* mutant oocytes (64.1% vs 24.0% in the mutant and WT, respectively) (Figure 6C).

### 2.6 D166G‐*Dpagt1* Mutant Oocyte Phenotypes Were Recapitulated by Oocyte‐Specific Knockout of *Dpagt1*

**Figure 7:**
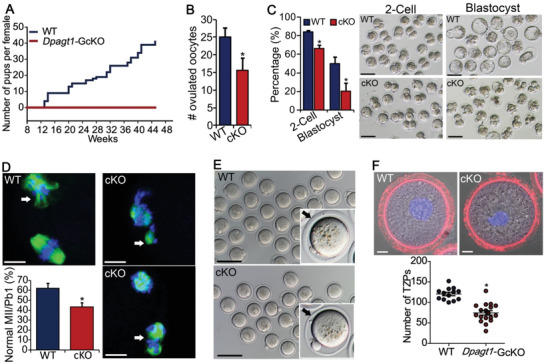
Recapitulation of the phenotypes in D166G‐*Dpagt1* mutant females by *Dpagt1*‐GcKOs. A) Cumulating number of pups born per female by the WT (*n* = 3) and *Dpagt1*‐GcKO (*n* = 7) females during the 9 month period of fertility test. B) Number of oocytes ovulated by WT and GcKO females after superovulation treatment. C) The rate (left bar graph) and representative micrograph (right panels) of 2‐cell and blastocyst formed by ovulated oocytes after IVF. Scale bars indicate 100 µm. D) Defective progression of meiosis to metaphase II by *Dpagt1*‐GcKO oocytes in vitro. Spindles are stained in green, and chromosome in blue. Scale bars indicate 10 µm. Arrows point to the extruded PB1. The bar graph shows the quantification of the rate of oocytes reaching normal MII. E) Light micrographs showing the thin and fragile ZP coat in ovulated MUT oocytes. Arrows point to ZP. Scale bars:100 µm. F) Phalloidin staining of TZPs (red) in immature GV‐oocytes. Quantification of the number of TZPs per oocyte is shown in the bottom bar graph. Scale bars indicate 10 µm. *p < 0.05, compared to WTs by Student's *t*‐test.

The phenotypes observed in D166G‐*Dpagt1* mutant oocytes could be caused by systemic malfunction of DPAGT1. To resolve this suspicion and to unravel the oocyte‐specific function of DPAGT1, cKO of *Dpagt1* in mouse oocytes was achieved by crossing *Dpagt1* ^loxp/loxp^ females with *Gdf9*‐Cre males in which the CRE recombinase was expressed in oocytes starting from the primordial follicle stage.^[^ ^31^ ^]^ *Dpagt1* was completely deleted in oocytes but not in other tissues tested (Figure S1E, Supporting Information). Phenotypic analysis of the oocyte development and female fertility was then carried out on the resultant *Dpagt1* ^loxp/loxp^; *Gdf9*‐Cre/+ (hereafter referred to as *Dpagt1*‐GcKO) females. *Dpagt1*‐GcKO female mice were completely infertile, with no pups produced during the 9 month breeding test period (**Figure** **7**A). The cKO females could ovulate but with fewer eggs than the WTs (Figure 7B). Moreover, the meiotic and developmental competence of the ovulated oocytes was compromised in the cKOs. After in vitro fertilization (IVF), the rate of formation of 2‐cell stage embryos was lower in *Dpagt1*‐GcKO oocytes (66.6%) than in the WT's (84.1%), and a more dramatic reduction in the development of the 2‐cell embryos into blastocysts was observed in the cKO (20.5% in the cKO vs 50.2% in the WT) (Figure 7C). Moreover, the percentage of mature oocytes at normal MII stage was significantly lower in *Dpagt1*‐GcKOs (43.5%) than in WTs (62.3%), with more *Dpagt1*‐GcKO oocytes bearing abnormal spindles and misaligned chromosomes (Figure 7D). *Dpagt1*‐GcKO oocytes also had thin and fragile ZP coat (Figure 7E), with fewer number of TZPs projecting to the oolema (Figure 7F).

### 2.7 Similar Alteration of Transcriptome in *Dpagt1*‐GcKO and D166G‐*Dpagt1* Mutant Oocytes

**Figure 8:**
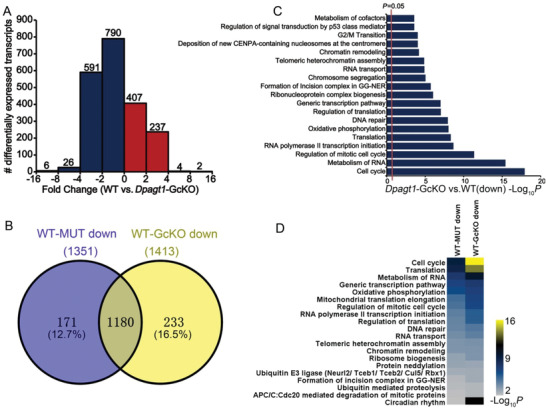
Similar alteration of transcriptome in *Dpagt1*‐GcKO and D166G‐*Dpagt1* oocytes. A) Distribution of the significantly changed transcripts in *Dpagt1*‐GcKO GV‐stage oocytes at various magnitudes of difference in expression levels detected by RNA‐Seq. The number of changed transcripts in each category of fold change is indicated above the bars. B) Venn diagram illustrating the relationship of the downregulated transcripts identified by RNA‐Seq in D166G‐*Dpagt1* and *Dpagt1*‐GcKO GV‐stage oocytes. WT–MUT: WT versus D166G‐*Dpagt1*; WT–GcKO: WT versus *Dpagt1*‐GcKO. The total number of downregulated transcripts is indicated in parentheses above the circles. C) Bar graph illustrating the enriched GO/KEGG terms or canonical pathways associated with the significantly downregulated transcripts in *Dpagt1*‐GcKO GV‐stage oocytes identified by RNA‐Seq. D) Heatmaps illustrating the enriched GO/KEGG terms or canonical pathways associated with the significantly downregulated transcripts in D166G‐*Dpagt1* and *Dpagt1*‐GcKO GV‐stage oocytes identified by RNA‐Seq.

To reveal that the molecular alteration resulted from oocyte‐specific deletion of *Dpagt1*, and to unravel to what extent the changes of D166G‐*Dpagt1* mutant oocyte transcriptome were contributed by oocyte loss of *Dpagt1*, transcriptomic changes in *Dpagt1*‐GcKO oocytes were analyzed and compared with that of D166G‐*Dpagt1* mutant oocytes. A total of 2063 transcripts were identified to be significantly changed in *Dpagt1*‐GcKO oocytes, as compared with the WT (**Figure** **8**A), of which more transcripts were found to be downregulated (1413 down and 650 up, respectively, in the cKO) (Figure 8A; Tables S6 and S7, Supporting Information). Comparison of the transcripts that were changed in D166G‐*Dpagt1* mutant oocytes with those changed in *Dpagt1*‐GcKO oocytes revealed high similarity between them (Figure S5A, Supporting Information). Of particular, 87.3% of the transcripts that were downregulated in D166G‐*Dpagt1* mutant oocytes were also found to be decreased in *Dpagt1*‐GcKO oocytes (Figure 8B). Gene enrichment analysis also revealed great similarity existed in the pathways and biological processes associated with the transcripts that were changed in *Dpagt1*‐GcKO and D166G‐*Dpagt1* mutant oocytes (Figure 8C,D; Figure S5B, Supporting Information).

## 3 Discussion

*Dpagt1* encodes dolichyl‐phosphate (UDP‐*N*‐acetylglucosamine) acetylglucosaminephosphotransferase 1 (GlcNAc‐1‐P transferase, DPAGT1) in the ER membrane that catalyzes the first step of *N*‐glycan biosynthesis, i.e., the transfer of GlcNAc‐P from UDP‐GlcNAc onto the carrier lipid dolichol‐phosphate (Dol‐P) to produce Dol‐PP‐GlcNAc.^[^ ^32^ ^]^ DPAGT1 is conserved between mouse and human, with 97% of identity in amino acid sequence. In mice, knockout of *Dpagt1* leads to lethality shortly after uterine implantation owing to widespread cell death, indicating the critical role of DPAGT1 in the maintenance of cell survival and embryo development.^[^ ^33^ ^]^ In human, over 26 missense variants of *DPAGT1* are identified in clinics, which either cause congenital myasthenic syndrome (DPAGT1‐CMS), or lead to a more severe multisystem syndrome, CDG‐Ij.^[^ ^34^, ^35^, ^36^, ^37^ ^]^ Given these critical roles of DPAGT1 in mice and humans, it is quite unexpected to find such a missense mutation of this gene (D166G‐*Dpagt1*) that mainly affects fertility in the female without overtly compromising the function of other systems. Moreover, the infertility‐causing defect in D166G‐*Dpagt1* mutant females appears most likely stemming from the oocyte since *Dpagt1*‐cKO in oocytes resulted in the similar, though more severe, phenotypes. Evidence that further buttresses this notion is that over 83% of the transcripts changed in D166G‐*Dpagt1* mutant oocytes were also altered in the *Dpagt1*‐cKO's. It is therefore tempting to speculate that the D166 residue of DPAGT1 functions specifically in oocytes. Therefore, the D166G‐*Dpagt1* missense mutant identified here by our mouse forward genetic screen provides a valuable animal model for addressing the role of DPAGT1, as well as the process of protein *N*‐glycosylation, in human female reproduction.

We found here that the D166G‐*Dpagt1* missense mutation did not affect either the expression or the localization of DPAGT1 within the oocyte. Then, what else could be the mechanism by which this particular D166G amino acid substitution predominantly interferes with the function of DPAGT1 in oocytes? The crystal structure of human DPAGT1 protein resolved recently provides immense aid to illuminating how the single amino acid substitution would change the function of DPAGT1 and lead to diseases.^[^ ^32^, ^38^ ^]^ By combining the structure analysis with mutagenesis and enzymatic activity assays, Dong et al. revealed that comparing with the loss of protein stability and RNA splicing defects, reduction of enzymatic activity is the most prevalent consequence of the reported disease‐causing missense point mutation of *DPAGT1* in human.^[^ ^32^ ^]^ Most interestingly, they found that human Leu168Pro and Tyr170Cys mutations cause significant loss of the function of DPAGT1, leaving its enzymatic activity less than 25%.^[^ ^32^ ^]^ Given that D164 (D166 in the mouse) is adjacent to Leu168 and Tyr170, which all lie on the ER side of the membrane and are at the top of the predicted Dol‐P binding site, and Leu168 and Tyr170 are involved in Dol‐P binding by DPAGT1,^[^ ^32^ ^]^ it is plausible that the D164G missense variant also causes the loss of DPAGT1 function by reducing its enzymatic activity. Dong et al. also revealed that the symptom onset and disease severity in DPAGT1‐CDG patients is determined by the residual levels of DPAGT1 activity, with no disease at 50% activity and most pronounced symptoms at 5–10% activity.^[^ ^32^ ^]^ In this regard, the D166G mutation identified here in the mouse is most likely a hypomorphic allele of *Dpagt1* gene. This mutation might cause the reduction of DPAGT1 activity to a threshold level that could impair oocyte development, but not to the point that is severe enough to affect the function of the other cell types. Alternatively, since DPAGT1 is known to form complex with another two ER membrane‐associated glycosyltransferases, namely, asparagine linked glycosylation (ALG) 13 and ALG14,^[^ ^39^ ^]^ the D166G missense mutation may affect the interaction of DPAGT1 with ALG13/ALG14 proteins, as well as other potential binding partners, which consequently impairs the normal function of the protein complex without necessarily affecting the enzyme activity of DPAGT1. All these possibilities await to be neatly verified by more sophisticated studies in the future. Nevertheless, in either case, the D166G missense mutation would eventually compromise protein *N*‐glycosylation. Indeed, we observed the significant alteration of the *N*‐glycoproteome and the hypo‐*N*‐glycosylation of a number of proteins in D166G‐*Dpagt1* mutant ovaries.

Mutation of *Dpagt1* caused defects in oocyte meiotic progression and preimplantation development, and distorted oocyte transcriptome. This suggests that DPAGT1 plays an indispensable role in ensuring the development of high‐quality oocytes by regulating the gene expression program of oocytes. Specifically, the downregulation of a subset of transcripts involved in “cell cycle and division” could be attributable to the meiotic defects observed in *Dpagt1* mutant oocytes. Of particular, PTTG1 is a known substrate of anaphase‐promoting complex (APC), which binds and inactivates the cohesin‐cleaving enzyme, separase, until the metaphase to anaphase transition.^[^ ^10^, ^11^ ^]^ Early onset of degradation of of PTTG1 in oocytes is associated with precocious extrusion of the first polar bodies.^[^ ^40^ ^]^ ESCOT2 is an acetyltransferase, and knockdown of its expression in mouse oocytes compromises spindle assembly, chromosome alignment, and kinetochore–microtubule attachments, and leads to precocious extrusion of the first polar body and increase of aneuploidy.^[^ ^30^ ^]^ Given these crucial roles of PTTG1 and ESCOT2 in the control of oocyte meiotic maturation, it is plausible to speculate that the complication of the first meiotic division in *Dpagt1* mutant oocytes is caused, at least in part, by the downregulation of the expression of these two factors. In addition, meiotic defects in *Dpagt1* mutant oocytes could be also partially caused by the downregulation of the expression of a group of genes responsible for oxidative phosphorylation and energy production. This is because that oocyte meiotic maturation is an active energy‐demanding process,^[^ ^41^ ^]^ and lower levels of ATP in oocytes are frequently found to be associated with defective spindle assembly and chromosome alignment.^[^ ^42^, ^43^ ^]^

Transcriptomic analysis also revealed the reduction of a number of transcripts in *Dpagt1* mutant oocytes that are involved in “oocyte acquisition of developmental competence” and “RNA metabolism and translation,” respectively, which could explain the compromised embryonic development phenotype observed in *Dpagt1* mutant oocytes. For example, *Dppa3/Stella*, *Npm2*, *H3f3b*, and *Khdc3/Fillia* belong to the maternal effect genes that are essential for preimplantation development.^[^ ^44^ ^]^ Depletion of the expression of these genes in oocytes causes either the abrogation of the normal transition of the one‐cell stage embryos to the two‐cell (i.e., *Npm2* and *H3f3b*),^[^ ^45^, ^46^, ^47^ ^]^ or defect of early embryonic development beyond the 2‐cell stage (i.e., *Dppa3/Stella*, *H3f3a/b*, and *Khdc3/Fillia*).^[^ ^48^, ^49^, ^50^ ^]^ Therefore, downregulation of the expression of these maternal‐effect genes, as well as others that are crucial for oocyte acquisition of developmental competence in *Dpagt1* mutant oocytes, would cumulatively lead to the impairment of the subsequent embryonic development. Moreover, given that selective degradation and translation of maternal transcripts during oocyte maturation are indispensable for oocyte completion of the first meiosis and oocyte‐to‐embryo transition,^[^ ^3^, ^51^, ^52^, ^53^, ^54^ ^]^ downregulation of the expression of transcripts involved in “RNA metabolism and translation” (e.g., *Dazl Eif4e*, and *Lsm5,6,10*, etc.) would also affect oocyte developmental competence.

Besides the genes that are known to be crucial for oocyte development, tanscriptomic analysis also revealed the downregulation of various transcripts encoding enzymes essential for synthesis of *N*‐glycans (i.e., *Alg13*, *Alg11*, *Dpm2*, *Ost4*, and *Ostc*), glycosaminoglycans (i.e., *Chsy1*), and glycophosphatidylinositol anchors (i.e., *Pigf*). Considering the critical roles of glycans in protein modification and the regulation of the diverse function of cells, reduction of the expression of the genes involved in synthesis of these specific classes of glycans in *Dpagt1* mutant oocytes could also affect the normal development of oocytes and eventually compromise their quality. Specifically, downregulation of *Alg13*, *Alg11*, *Dpm2*, *Ost4*, and *Ostc* could contribute to the overall protein hypo‐*N*‐glycosylation observed in *Dpagt1* mutant oocytes. *Chsy1* encodes chondroitin sulfate synthase 1, one of six glycosyltransferases that catalyze the biosynthesis of chondroitin sulfate proteoglycans (CSPGs).^[^ ^55^ ^]^ CSPGs, such as serum protein inter‐*α*‐trypsin inhibitor (I*α*I) and granulosa cell‐derived versican, are present in follicular fluid, and are involved in regulation of cumulus‐oocyte complexes (COCs) maturation by remodeling the hyluronan‐rich ECM formed around the COCs during ovulation.^[^ ^56^, ^57^, ^58^, ^59^, ^60^ ^]^ Although the specific contribution of oocyte‐derived CSPGs to the follicular microenvironment remains largely unclear, the presence of the critical enzymes for CSPG biosynthesis (e.g., CHSY1) in oocytes suggests a role in oocyte and follicle development. In this regard, downregulation of the expression *Chsy1* in *Dpagt1* mutant oocytes would also compromise oocyte developmental competence. *Pigf* encodes phosphatidylinositol glycan anchor biosynthesis class F, which is involved in glycosylphosphatidylinositol (GPI)‐anchor biosynthesis.^[^ ^61^ ^]^ Abrogation of GPI anchor biosynthesis in oocytes causes fertilization failure and female infertility,^[^ ^62^ ^]^ which indicates that the presence of GPI‐anchored proteins on oocyte surface is critical for oocyte development. Moreover, JUNO is the best‐known oocyte‐expressed GPI‐anchored protein that binds with sperm protein IZUMO1, and is indispensable for sperm–egg fusion during fertilization^[^ ^63^ ^]^. It is therefore plausible to predict that the reduction of *Pigf* expression contributes to the defective development of *Dpagt1* mutant oocytes.

ZP proteins are bona fide glycoproteins. This notion is well reinforced by observations made here that ZP proteins were hypo‐*N*‐glycosylated in *Dpagt1* mutant oocytes, and the ZP structure in both *Dpagt1* mutant and *Dpagt1*‐GcKO oocytes is severely flawed. Although oocytes of both allele females have a thin and fragile ZP coat, they can be fertilized normally. This suggests that protein *N*‐glycosylation of ZP proteins is not necessary for sperm binding and the eventual fertilizing of the eggs, but is instead crucial for proper assembly of the ZP structure. Given the viscous nature of the zona matrix, ZP is considered to serve to stabilize the gap junctions established between the microvilli emanating from the oocyte and TZPs from the companion granulosa cells that traverse the ZP coat.^[^ ^64^ ^]^ The increased fragility of zona observed here in both *Dpagt1* mutant and *Dpagt1*‐GcKO oocytes will probably compromise the formation of gap junctions between the oocyte and granulosa cells. Indeed, we observed here that the number of TZPs within the ZP matrix is significantly reduced in both *Dpagt1* mutant and *Dpagt1*‐GcKO oocytes, indicating the impairment of gap junctions. Because gap junctions are the fundamental physical basis for bilateral communication between the oocyte and the companion granulosa cells, which are essential for supporting the development of both compartments,^[^ ^65^, ^66^, ^67^ ^]^ the distortion of ZP structure and the consequent reduction of TZPs would undoubtedly interfere granulosa cell proliferation and follicle growth. In accord with this predication, we observed the evident defect of follicular development beyond the late secondary stage in *Dpagt1* mutant ovaries.

It is interesting to note that while we were working on this project, Kanaki et al. reported very recently that they have successfully identified the ortholog of human *DPAGT1* in nematode *Caenorhabditis elegans*.^[^ ^68^ ^]^ By inhibiting the activity of DPAGT1 through the RNAi approach, they demonstrated that DPAGT1 is also indispensable for oogenesis and oocyte‐to‐embryo transition in *C. elegans*.^[^ ^68^ ^]^ These findings in *C. elegans* are in accord with what we have revealed here in mice, thus indicating that the role of DPAGT1, as well as that of protein *N*‐glycosylation by inference, is evolutionarily conserved from invertebrates to mammals during oogenesis.

## 4 Conclusions

This study uncovers an oocyte‐specific role of DPAGT1 in the regulation of oocyte gene expression and acquisition of meiotic and developmental competence in mammalian species. Although specific mutations of *DPAGT1* that affect human fertility have not been identified, it is anticipated that with the rapid development of the cost‐effective whole genome‐sequencing techniques, more and more novel disease‐causing human mutations will be identified for genes involved in the protein *N*‐glycosylation pathway, particularly those for *DPAGT1*. The missense mutation of *Dpagt1* and the oocyte‐specific function of DPAGT1 protein revealed here will certainly be insightful for deciphering the potential role of the human ortholog of *Dpagt1* in human oogenesis, as well as the contribution of protein *N*‐glycosylation to human female fertility control.

## 5 Experimental Section

##### Laboratory Mice

All mice used for experiments were bred and raised under the standard conditions in the investigator's colony at the Animal Core Facility of Nanjing Medical University (Nanjing, P. R. China). Mouse experimental procedures and protocols were approved by the Institutional Animal Care and Use Committee of Nanjing Medical University (Approval number: IACUC14030156), and were conducted in accordance with the institutional guides for the care and use of laboratory animals.

D166G‐*Dpagt1* missense point‐mutant mice were created by the ENU‐mutagenesis‐based Translational Vision Research Models (TVRM) program at the Jackson Laboratory (Bar Harbor, ME, USA) as previously described.^[^ ^69^ ^]^ These D166G‐*Dpagt1* mutants, along with the mice carrying a floxed allele of *Dpagt1* (*Dpagt1* ^tm2Jxm^/J, JAX stock# 006887, hereafter referred to as *Dpagt1* ^F/F^) and the Tg(*Gdf9*‐icre)5092Coo (hereafter referred to as *Gdf9*‐Cre) allele, respectively, were imported from the Jackson Laboratory to the investigator's laboratory at Nanjing Medical University. All of these mice were maintained on identical C57BL/6J genetic background, and were genotyped by PCR using primers as shown in Table S1 in the Supporting Information. To create the oocyte‐specific *Dpagt*‐KO mice, *Dpagt1* ^F/F^ females were first crossed with *Gdf9*‐Cre/+ males, which produces *Dpagt* ^F/+^;*Gdf9*‐Cre/+ progenies. The resulted *Dpagt* ^F/+^;*Gdf9*‐Cre/+ males were then crossed with *Dpagt1* ^F/F^ females to make *Dpagt1* ^F/F^;*Gdf9*‐Cre/+ mice. The colonies were then routinely maintained in a breeding scheme of *Dpagt1* ^F/F^ females crossing with *Dpagt1* ^F/F^;*Gdf9*‐Cre/+ males to produce either *Dpagt1* ^F/F^ or *Dpagt1* ^F/F^;*Gdf9*‐Cre/+ cKO (hereafter referred to as *Dpagt1*‐GcKO) females for experiments. Normal wildtype ICR mice were purchased from the Animal Core Facility of Nanjing Medical University, while the normal C57BL/6JXDBA2 (B6D2) F1 mice were produced at the investigator's own colony.

##### Fine Mapping and Positional Cloning

Phenotypically affected C57BL/6J (the parental strain that was mutagenized) mice were mated with DBA/2J to take advantage of the higher degree of polymorphism between these two strains. The resulting F1 offspring were intercrossed to generate the F2. The resulting progeny were then phenotyped, and linkage‐based (linkage was implicated by the association of phenotype with homozygosity for the parental C57BL/6J strain that was mutagenized) whole genome scan for the candidate chromosome harboring the ENU mutation and fine mapping the candidate region of the mutation were then carried out using ≈80 robust simple sequence length polymorphic (SSLP) markers distributed throughout the genome and known to differ between C57BL/6J and DBA/2J. Final identification of the mutation within the candidate gene was done by PCR amplification of the desired genomic region, as well as the cDNA of the candidate gene, and sequencing the PCR products using standard procedures.

##### Fertility Test

Fertility test was carried out by mating the 8 week old female mice of different genotypes with normal adult B6D2 F1 males with proven fertility for up to 9 months. The number of pups for each litter was recorded at birth, and the average accumulating number of pups per female was calculated at the end of the fertility test.

##### Oocyte Isolation and In Vitro Maturation, Fertilization, and Embryo Culture

Oocyte collection and culture was carried out in bicarbonate‐buffered MEM medium (Thermo Fisher Scientific Inc., Waltham, MA, USA) with Earles’ salts, supplemented with 75 µg mL^−1^ penicillin G, 50 µg mL^−1^ streptomycin sulfate, 0.23 × 10^−3^ m pyruvate, and 3 mg mL^−1^ bovine serum albumin. Oocyte and embryo were cultured at 37 °C and 100% humidity in an Eppendorf New Brunswick Galaxy170R incubator (Hamburg, Germany) infused with 5% O_2_, 5% CO_2_, and 90% N_2_. All the supplements of the culture medium were purchased from Sigma‐Aldrich, USA.

For studying the ability of oocytes to resume and complete the first meiosis, and the kinetics of oocyte meiotic progression, fully grown oocytes (FGOs) were isolated from equine chorionic gonadotropin (eCG, Ningbo A Second Hormone Factory, Cixi, China)‐primed (46 h) 23 day old female mice, and were matured in culture as described previously.^[^ ^70^ ^]^ Oocyte resumption and completion of the first meiosis, as manifested by GVB and first polar body (PB1) extrusion, respectively, was recorded during the culture. Additionally, oocytes were collected after 8 and 14 h of IVM for IF analysis of spindle morphology and chromosome alignment, SAC activation and kinetochore–microtubule attachment at MI stage, and euploidy status at MII.

For IVF, oocytes were collected from oviductal ampulla of the mice that were initially primed with eCG for 48 h followed by human chorionic gonadotropin (hCG, Ningbo, China) for 14 h. The ovulated oocytes were then fertilized with normal sperm isolated from B6D2F1 adult males. The formation of 2‐cell stage embryos was recorded 24 h after IVF; thereafter, they were transferred into KSOM medium for further development to the blastocyst stage. The formation of 4‐cell, morula, and blastocyst stage embryos were scored on day 1, day 2, and days 3–5 of the culture, respectively.

##### Spinning Disc Confocal Microscopy of Live Oocytes

To study the kinetics of oocyte meiotic maturation through live imaging, WT‐ and D166G‐*Dpagt1* mutant GV‐stage FGOs were cultured in 10 µL drop of maturation medium covered with washed mineral oil in a 30 mm petri dish, and imaged under an Andor Spinning disk confocal microscope (Andor Technology Ltd, Belfast, UK) as previously described in details.^[^ ^70^ ^]^ The DIC bright images of the live oocytes were captured in a 1 h interval for up to 14 h. The static images showing the kinetics of oocyte extrusion of PB1 were exported using the image processing software associated with the Andor spinning disk confocal microscope.

##### Ovarian Histology and Immunofluorescence

These were carried out following the same procedure as previously described. ^[^ ^68^ ^]^ Briefly, ovaries from eCG‐primed (46 h) 23 day old, and eCG‐unprimed 21 day old female mice were fixed in Bouin's fixative, embedded in paraffin, and serially sectioned at 5 µm thickness. The ovarian sections were then stained with periodic acid‐Schiff reagent (Sigma‐Aldrich, USA) and Lillie‐Mayer hematoxylin, and follicles at various stages of development were counted on every third sections throughout the whole ovary. For IF staining, freshly isolated ovaries from eCG‐unprimed 21 day old female mice were fixed in 4% paraformaldehyde (PFA, Sigma‐Aldrich, USA) prepared in PBS at 4 °C overnight, and 5 µm thick paraffin sections were then prepared. Sections were incubated with the primary antibodies to KI‐67 (catalog no. ab16667; 1:200, Abcam, USA), ZP1 (catalog no. sc‐32751; 1:500, Santa Cruz, USA), ZP2 (catalog no. sc‐32752; 1:500, Santa Cruz, USA), and ZP3 (catalog no. sc‐398359; 1:500, Santa Cruz, USA), respectively, followed by Alexa flour 594‐ or 488‐conjugated secondary antibodies (Thermo Fisher Scientific, USA), and were imaged under an LSM 700 confocal laser scanning microscope (Zeiss, Germany).

##### Immunostaining and Confocal Microscopic Imaging of Oocytes and Blastocysts

Both oocyte and blastocyst samples were fixed in 4% PFA in PBS for 30 min at room temperature, followed by permeabilization and blocking for 1 h in PBS containing 0.1% Triton‐100 (Sigma) and 10% fetal bovine serum (FBS) (Thermo Fisher Scientific). The samples were subsequently incubated with primary antibodies (4 °C, overnight) and Alexa flour 594/488‐conjugated secondary antibodies (room temperature, 30 min), respectively, and counterstained with Hoechst 33342 for 20 min. The samples were finally mounted on glass slides and examined under an LSM 700 laser scanning confocal microscope (Carl Zeiss). Images for different groups of samples in the same experiment were taken under the same parameters. The primary antibodies used for staining of oocyte spindles and SAC were monoclonal anti‐*β*‐tubulin antibody produced in mouse (catalog no. T4026; 1:500, Sigma) and sheep polyclonal BubR1 antibody (catalog no. ab28193; 1:200, Abcam, USA). Blastocysts were stained with OCT4 (catalog no. ab28193, 1:500, Abcam,) and CDX2 (catalog no. CA 94538, 1:500, BioGenex, USA) for distinguishing the cells of ICM and TE, respectively.

##### Assessment of Kinetochore–Microtubule Attachment and Euploidy in Oocytes

Oocytes that were matured in vitro for 8 h were subjected to cold treatment on ice for ≈10 min, and then fixed in 2.4% PFA for 30 min at room temperature. These oocytes were subsequently immunostained for kinetochores and microtubules using the human anticentromere antibodies (catalog no.15‐234, 1:500, Antibodies Incorporated, USA) and mouse monoclonal anti‐*β*‐tubulin antibody (catalog no.T4026; 1:500, Sigma), respectively. For analysis of euploidy status, mature oocytes were briefly (30 s) incubated in Tyrode's buffer (pH 2.5) at 37 °C to have the zona pellucida removed. The oocytes were then fixed on a glass slide in a drop of 1% PFA that was prepared with distilled H2O and containing 0.15% Triton X‐100 and 3 × 10^−3^ m dithiothreitol, and air‐dried at room temperature. The spread chromosomes were finally incubated with the human anticentromere antibody (1:500, Antibodies Incorporated) at 4 °C overnight, followed by incubation with Alexa Fluor 594‐conjugated donkey anti‐Human IgG secondary antibody (Thermo Fisher Scientific), and counterstained with Hoechst 33342 (Sigma). All the specimens were examined and imaged using a laser scanning confocal microscope (LSM 700, Carl Zeiss).

##### Transmission Electron Microscopy

COCs isolated from large antral follicles were fixed in 2.5% glutaraldehyde (Sigma) for 2 h at 4 °C, followed by three washes in PBS with each for 15 min. These COCs were briefly stained with eosin (Sigma) for 15 s to make them easily identifiable, and were then embedded in 1.5% agarose. The agarose gel cube (2 × 2 × 2 mm) containing COCs was fixed overnight in 2.5% glutaraldehyde, and sent to the Biomedical Imaging Core Facility at Nanjing Medical University for transmission electron microscopic analysis using a standard protocol.

##### Examination of Implantation Sites

Adult (8–9 weeks old) females were mated with fertile wild type males to induce pregnancy. The day on which vaginal plug formed was designated as day 1 postcoitus. The number of IS on day 5 postcoitus was examined after staining with 0.1% Chicago sky blue solution (Sigma) via intravenous administration as described previously.^[^ ^71^ ^]^ The IS number on day 8 was evaluated by directly counting the visualizable fetuses in the uterine horns.

##### Apoptosis Assay by TUNEL Staining

To detect cellular apoptosis in the ovaries, 4% PFA fixed ovarian paraffin sections were prepared as stated in the preceding part of the Experimental Section. TUNEL staining was then performed on these sections using the TMR red in situ cell death detection kit (Sigma), as described in details previously. ^[^ ^72^ ^]^ DNA was counterstained with Hoechst 33342 (Sigma). Images were taken using a LSM700 confocal microscope (Zeiss), and the TUNEL positively stained cells within the follicles were counted.

##### RNA‐Seq and qRT‐PCR Analyses

RNA‐Seq analysis was carried out on triplicate samples of WT‐, D166G‐*Dpagt1*‐, and *Dpagt1*‐GcKO oocytes as described previously, ^[^ ^68^ ^]^ with each sample having 20 GV‐stage FGOs. The differentially expressed transcripts were calculated using default parameter of cuffdiff (v2.2.1), and the significantly changed transcripts were defined by the criteria of FDR *q* < 0.05 and |fold change| > 2. The RNA‐Seq data are deposited in the Gene Expression Omnibus with the dataset accession number GSE140609. Bioinformatic analysis of differentially expressed transcripts was conducted using Metascape (http://metascape.org), a gene annotation and analysis resource, following the online instruction provided by the web developer. For qRT‐PCR analysis, total RNA was isolated from oocyte samples using the RNeasy Micro Kit (Qiagen), and reversed transcribed by the QuantiTect Reverse Transcription Kit (Qiagen). SYBR Green‐based quantitative real‐time PCR analysis was then carried out using primer pairs shown in Table S1 in the Supporting Information. Relative changes in mRNA levels between WT‐ and D166G‐*Dpagt1* or *Dpagt1*‐GcKO oocytes were analyzed by the 2^−ddCt^ method using *Rpl19* as internal control.

##### *N*‐Glycoproteomic Analysis

High‐accuracy mass spectrometry‐based *N*‐glycoproteomic analysis was carried out on triplicate sets of samples of WT‐ and D166G‐*Dpagt1* mutant ovaries using a standard protocol as described previously ^[^ ^73^ ^]^ at the proteomics core facility of the Institute of Biomedical Sciences at Fudan University (Shanghai, China). Briefly, 10 pairs of ovaries per group of each genotypes were lysed and protein was extracted and quantified. Then, 300 µg of each protein sample was digested with Trypsin, followed by enrichment with lectin solution containing a combination of ConA, WGA, and RCA120 (Sigma). The enriched peptides were then deglycosylated with PNGase F, purified, and subjected to LCMS analysis on an Easy‐nLC 1200 and Fusion Lumos system (Thermo Scientific). The LC‐MS/MS data were analyzed using Proteome Discoverer software (version 2.3.0.523) provided by Thermo Fisher Scientific. Statistical analysis was carried out on the quantified data using Perseus package, and significant difference was defined by the criteria of *p* < 0.01 and fold change >2. The mass spectrometry proteomics data have been deposited to the ProteomeXchange Consortium via the PRIDE partner repository with the dataset identifier PXD018531.

##### Cloning and Overexpression of Dpagt1

Both WT‐ and D116G‐*Dpagt1* ORF were amplified by PCR using the cDNA derived from the WT‐ and D116G‐*Dpagt1* mutant oocytes, and cloned into the pCMV6‐C‐3DDK vector (Origene, USA). The correct sequences of the inserts were verified by Sanger sequencing. mRNAs were then synthesized and microinjected into the WT‐immature GV‐stage oocytes as described previously.^[^ ^70^ ^]^ Expression of both the WT‐ and D116G‐mutant DPAGT1‐3DDK fusion protein was then examined by WB and IF analyses using the anti‐FLAG/DDK antibody.

##### Western Blot Analysis

Oocytes were lysed in 2× Laemmli sample buffer, and heated at 108 °C for 5 min. The denatured proteins were resolved on SDS‐PAGE and transferred onto polyvinylidene difluoride (PVDF) membranes for probing the proteins under examination. The expression of *β*‐actin (ACTB) served as internal control of each sample. The following primary antibodies were used: anti‐*β*‐actin (ACTB) antibody (catalog no. A1978, 1:5000, Sigma), anti‐Securin/PTTG1 antibody (catalog no. ab79546, 1:500, Abcam), ESCO2 antibody (catalog no. A301‐689A‐M, 1:500, Bethyl Laboratories, Inc, USA), ZP1 (catalog no. sc‐32751; 1:500, Santa Cruz, USA), ZP2 (catalog no. sc‐32752; 1:500, Santa Cruz, USA), and Monoclonal anti‐FLAG (DDK) M2 antibody (catalog no. F3165; 1:10000, Sigma, USA).

##### Statistical Analysis

All statistical analyses were performed using Graphpad Prism software (Graphpad software, Inc, La Jolla, CA, USA). Student's *t*‐tests were used to compare differences between two groups, with *p* < 0.05 defined as significantly different. For experiments involving more than two groups, differences between groups were compared by one‐way ANOVA followed by Tukey's Honestly Significant Difference (HSD), and *p* < 0.05 was assigned to significantly different. Data were presented as mean ± standard error of mean (SEM) of at least three independent experiments.

## Conflict of Interest

The authors declare no conflict of interest.

## Author Contributions

Y.‐Q.S. conceived the study; H.L., L.Y., Y.T., X.F., J.G., C.Z., and L.S. performed the research; H.L. and Y.‐Q.S. analyzed the data; and H.L. and Y.‐Q.S. wrote the paper.

## Supporting information

Supporting Information

Supplemental Table 2

Supplemental Table 3

Supplemental Table 4

Supplemental Table 5

Supplemental Table 6

Supplemental Table 7
