# Supplementary material for: DPAGT1‐Mediated Protein N‐Glycosylation Is Indispensable for Oocyte and Follicle Development in Mice
Source: Adv Sci (Weinh). 2020 Jun 3;7(14):2000531. doi: 10.1002/advs.202000531 (PMC7375233; doi:10.1002/advs.202000531)
Supplement: Supplementary file 1 — Supporting Information [file ADVS-7-2000531-s001.pdf]

Copyright WILEY-VCH Verlag GmbH & Co. KGaA, 69469 Weinheim, Germany, 2020.

Supporting Information

Title : **DPAGT1-Mediated Protein N-Glycosylation Is Indispensable for Oocyte and Follicle Development in Mice**

*Hui Li, Liji You, Yufeng Tian, Jing Guo, Xianbao Fang, Chenmin Zhou, Lanying Shi, Patsy Nishina, and You-Qiang Su**

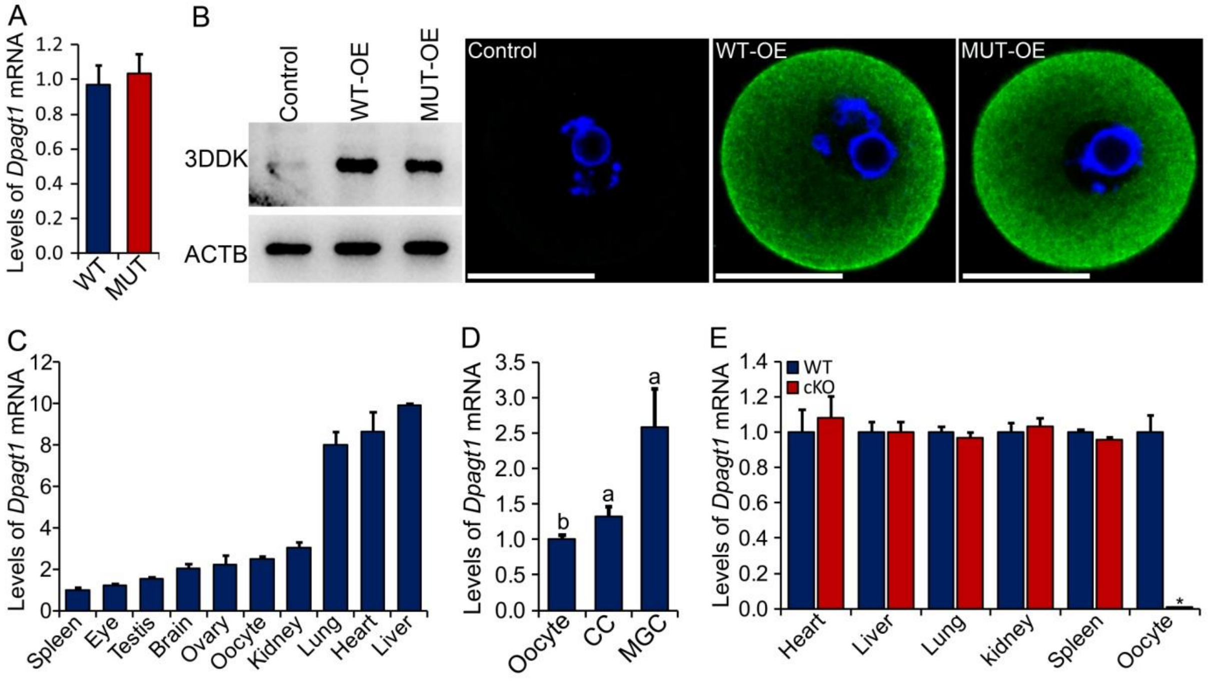

Figure S1. Expression and localization of *Dpagt1* mRNA or protein in oocytes, granulosa cells, and multiple tissues. A) Comparison of *Dpagt1* mRNA expression in oocytes between wild type (WT) and D166G-*Dpagt1* mutant (MUT) mice. B) Western blot- (left panel) and immunofluorescence- (right panels) detecting the expression of exogenous 3DDK-tagged wild type (WT) and D166G-*Dpagt1* mutant mRNA microinjected into WT oocytes. Oocytes injected with water serve as control. OE, overexpression. Scale bars indicate 50µm. C) Expression of *Dpagt1* mRNA in oocytes and various tissues. D) Comparison of *Dpagt1* mRNA expression between the oocyte and the somatic compartment within large antral follicles. CC, cumulus cell; MGC, mural granulosa cells. Bars with different letters denote significant different, p <0.05. E) Comparison of *Dpagt1* mRNA expression in oocytes and the indicated tissues between WT and *Dpagt1*-GcKO (GcKO) mice. * p < 0.05, compared with WTs.

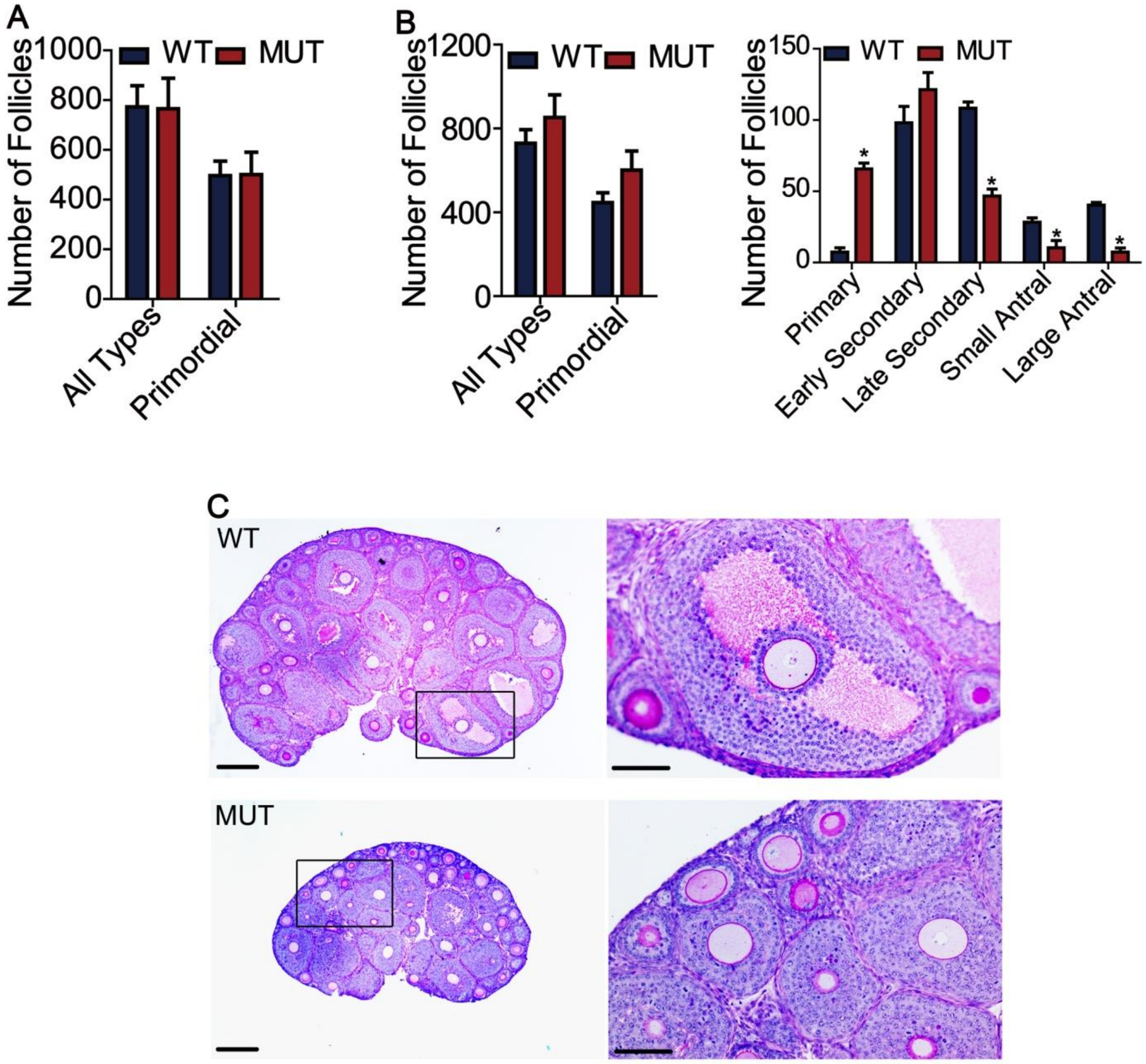

Figure S2. Assessment of changes of follicular development in ovaries of D166G-*Dpagt1* mutant (MUT) mice. A) Quantification of the number of total follicles and primordial stage follicles in WT and MUT ovaries from PMSG-unprimed 21d-old mice. B) Quantification of the number of total follicles and follicles at various developmental stages in WT and MUT ovaries from 23d-old PMSG-primed mice. C) Representative micrographs of PAS-stained WT and MUT ovarian sections from 23d-old PMSG-primed mice. Magnified views of the boxed areas are shown in the right panels adjacent to the original graphs. Scale bars indicate 100µm.

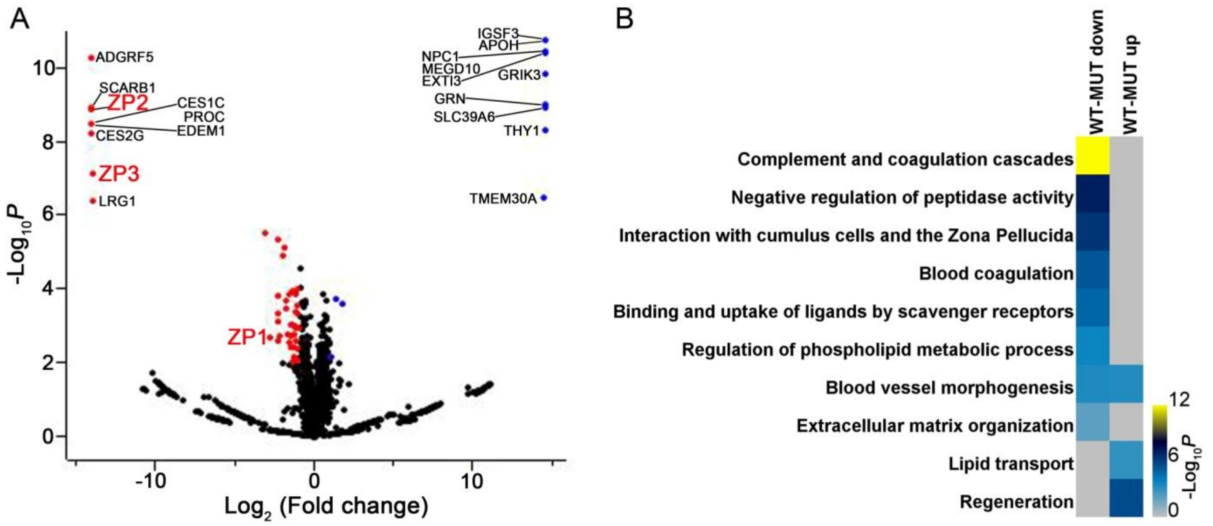

Figure S3. N-glycoproteomic analysis of the ovaries of wild type (WT) and D166G-*Dpagt1* mutant (MUT) female mice. A) Volcano plot illustrating the proteins with their N-glycosylation significantly changed in MUT ovaries as compared with the WTs. Red spots denote the proteins with their N-glycosylation downregulated in MUT ovaries, while the blue ones indicate the proteins with their N-glycosylation upregulated. B) Heatmaps illustrating the enriched GO/KEGG terms or canonical pathways associated with the proteins with their N-glycosylation either down- (WT-MUT down) or up- (WT-MUT up) regulated in D166G-*Dpagt1* mutant (MUT) ovaries.

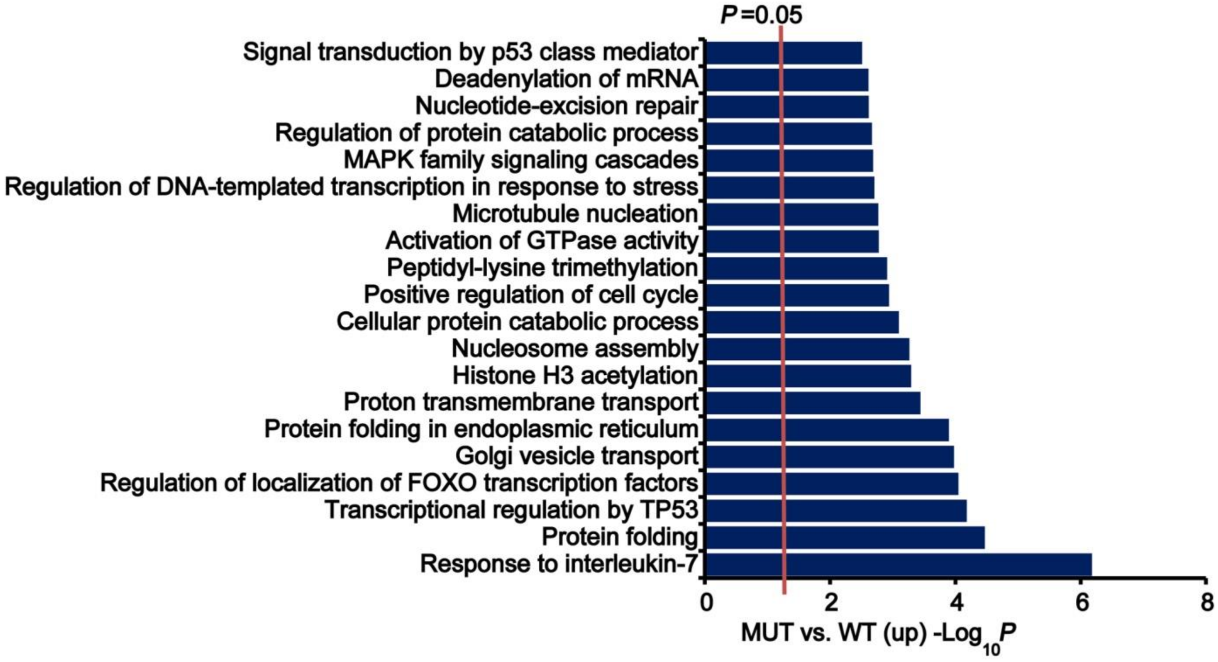

Figure S4. GO/KEGG terms or canonical pathways associated with the significantly upregulated transcripts in D166G-*Dpagt1* mutant (*MUT)* GV-stage oocytes identified by RNA-seq.

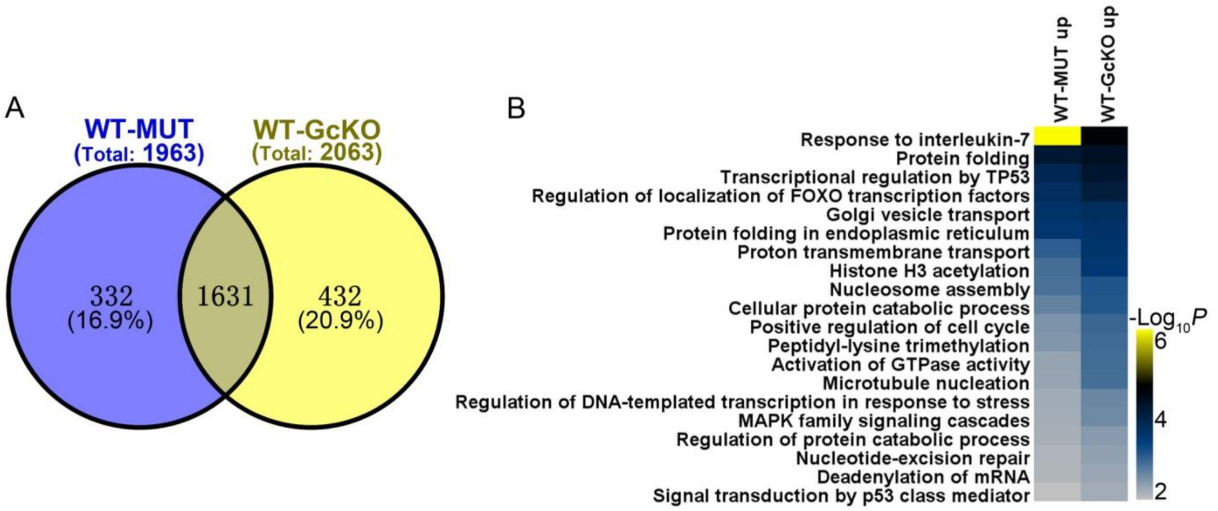

Figure S5. Similar alteration of transcriptome in *Dpagt1*-GcKO and D166G-*Dpagt1* oocytes. A) Venn diagram illustrating the relationship of the total changed transcripts identified by RNA-seq in D166G-*Dpagt1* (MUT) and *Dpagt1*-GcKO (GcKO) GV-stage oocytes. WT–MUT: WT vs D166G-*Dpagt1* mutant; WT–GcKO: WT vs *Dpagt1*-GcKO. The total number of changed transcripts is indicated in parentheses above the circles. B) Heatmaps illustrating the enriched terms GO/KEGG terms or canonical pathways associated with the significantly upregulated transcripts in D166G-*Dpagt1* (MUT) and *Dpagt1*-GcKO (GcKO) GV-stage oocytes identified by RNA-seq.

Table S1. Sequence of the primes used in this study. "F" and "R" indicate forward and reverse primers, respectively. Primers that are not specified for genotyping or cloning are designated for qRT-PCRs.

Name	Sequence (5′-3′)
Alg13 F	CTCTGGCATTGGTGCGAGC
Alg13 R	CGAGGATCTGAACGCAGTCG
Anapc16 F	CACCTACCCGAAAGGAGCTG
Anapc16 R	CCTGTTTAAGCGTGGATGCC
Cenpq F	AAGTCATGCAAAGCATCTGTCC
Cenpq R	TGCCAGGTTGTTCTCTTGCTG
Dpagt1 F	TCGTGGAGGAGCAGTGTAAGG
Dpagt1 R	TCAGCAAACCCCAGGAAGAT
Esco2 F	TCCCTCAGGTGCAGTAGTTTC
Esco2 R	TCTTCAGGTCTTTTTGTGTTCACTG
Ldhb F	GCCATCAGCATTCTGGGAAAG
Ldhb R	TCGGCCACAATTTTCGGAGT
Lsm10 F	TGGACCTTCGGGATGAGAGT
Lsm10 R	CAGCGGTCGGTATAGGTGAC
Npm2 F	CCATGGTCACTGTGTCAGGTA
Npm2 R	CATAACATTCCAGGCCACTGAG
Orc6 F	GCACTACTCTCAGCATGCAAAAT
Orc6 R	TGTTAATCTGCTGCCCAATCTT
Ostc F	TGTACGCCCTGGTAGTGGTA
Ostc R	TGCCCATGTTCATCGGTCAT

Paip1 F	CAGGCCACGTCTATCCCAAAT
Paip1 R	CATACTCCGTCCTGCACCTT
Pttg1 F	CGCCGTTTGGCATCTAAGGA
Pttg1 R	GACTCGAGGCGTTGAAACCT
Rpl19 F	TCAGGCTACAGAAGAGGCTTGC
Rpl19 R	ATCAGCCCATCCTTGATCAGC
Gdf9-iCre F_genotyping	TCTGATGAAGTCAGGAAGAACC
Gdf9-iCre R_genotyping	GAGATGTCCTTCACTCTGATTC
Dpagt1-gf1 F_cKO genotyping	TGGGGTTTGCTGATGATGT
Dpagt1-gR1 R_cKO genotyping	GATTGCTGGGATTTGAACTC
Dpagt1-loxp F_cKO genotyping	GGTGTGCTTCCACACTCTCA
Dpagt1-loxp R_cKO genotyping	TGACACCGCTAGTTCTGTGG
3DDK-Dpagt1 F_cloning	AGATCTGCCGCCGCGATCGCATGTGGGCCTTCCCGGAG
3DDK-Dpagt1 R_cloning	GCGGCCGCGTACGCGTGACATCATAGAAGAGTCGGAC

Captions for Tables S2 to S7

Table S2. List of the proteins with their N-glycosylation downregulated in D166G-*Dpagt1* mutant ovaries.

Table S3. List of the proteins with their N-glycosylation upregulated in D166G-*Dpagt1* mutant ovaries.

Table S4. List of the transcripts that are downregulated in D166G-*Dpagt1* mutant oocytes as compared with the WT-oocytes.

Table S5. List of the transcripts that are upregulated in D166G-*Dpagt1* mutant oocytes as compared with the WT-oocytes.

Table S6. List of the transcripts that are downregulated in in *Dpagt1*-GcKO oocytes as compared with the WT-oocytes.

Table S7. Table S4. List of the transcripts that are upregulated in in *Dpagt1*-GcKO oocytes as compared with the WT-oocytes.
